# Non-infectious sternal dehiscence after coronary artery bypass surgery

**DOI:** 10.1186/s13019-022-02015-1

**Published:** 2022-10-03

**Authors:** Martin Silverborn, Leon Arnar Heitmann, Nanna Sveinsdottir, Sigurjon Rögnvaldsson, Tomas Thor Kristjansson, Tomas Gudbjartsson

**Affiliations:** 1 Department of Cardiothoracic Surgery, Landspitali University Hospital, Reykjavík, Iceland; 2 Department of Cardiothoracic Surgery, Sahlgrenska University Hospital, Gothenburg, Sweden; 3 Faculty of Medicine, University of Iceland, Reykjavík, Iceland

## Abstract

**Introduction:**

Non-infectious sternal dehiscence (NISD) is a known complication following coronary artery bypass grafting (CABG), with previous studies estimating an incidence of 0.4–1% of surgeries. We aimed to study the incidence of NISD together with short- and long-term outcomes in a whole-nation cohort of patients.

**Materials and methods:**

A retrospective study on consecutive CABG patients diagnosed with NISD at Landspitali from 2001 to 2020. Patients diagnosed with infectious mediastinitis (n = 20) were excluded. NISD patients were compared to patients with an intact sternum regarding patient demographics, cardiovascular risk factors, intra- and postoperative data, and estimated overall survival. The median follow-up was 9.5 years.

**Results:**

Twenty out of 2280 eligible patients (0.88%) developed NISD, and the incidence did not change over the study period (p = 0.98). The median time of diagnosis was 12 days postoperatively (range, 4–240). All patients were re-operated using a Robicsek-rewiring technique, with two cases requiring a titanium plate for fixation. Patients with NISD were older, had a higher BMI and EuroSCORE II, lower LVEF, and more often had a history of COPD, MI, and diabetes compared to those without NISD. Length of stay was extended by 15 days for NISD patients, but short and long-term survival was not statistically different between the groups.

**Conclusions:**

The incidence of NISD was low and in line with previous studies. Although the length of hospital stay was extended, both short- and long-term survival of NISD patients was not significantly different from patients with an intact sternum.

## Introduction

Median sternotomy is by far the most common approach to access the heart during open heart surgery, as it gives excellent exposure to the heart and its central vessels. However, complications with healing after the sternotomy can occur and are often divided by their etiology into infectious or non-infectious. To the latter group belongs dehiscence of the sternum, defined as non-infectious separation and/or movement of the sternum, with an incidence in the literature ranging from 0.4 to 1% [1, 2]. By definition, non-infectious sternum dehiscence (NISD) does not include cases where dehiscence is a part of or causing a deep sternal wound infection (DSWI), also called post-sternotomy mediastinitis; one of the most serious complications following open heart surgery [3–5].

NISD usually presents with secretion and increased pain from the sternotomy wound, often leading to impaired respiration and pulmonary complications such as pneumonia [6]. If diagnosed late, the risk of NISD developing into DSWI is imminent [7, 8]. Although NISD is far less studied than DSWI, with most studies including a limited number of patients, several pre- and postoperative risk factors of NISD have been reported in the literature. These include a high body mass index (BMI) [6, 9], diabetes mellitus [10], chronic obstructive pulmonary disease (COPD) [6, 10], and a high New York Heart Association (NYHA) class [11]. However, previous studies almost solely focus on early complications and 30-day mortality, but long-term survival remains inadequately addressed.

Therefore, we aimed to describe the incidence and short-term outcomes of these patients, as well as long-term survival, in a well-defined whole-nation cohort of coronary artery bypass grafting (CABG) patients.

## Materials and methods

### Data curation

This nationwide retrospective cohort study included consecutive patients that underwent first-time isolated CABG with or without cardiopulmonary bypass (CPB) in Iceland from January 1, 2001, to December 31, 2020, at Landspitali National University Hospital; the only hospital in Iceland performing open heart surgery. In total, 2300 patients undergoing CABG for any indication were identified and considered for inclusion.

Clinical data were derived from two internal surgical registries at Landspitali. Firstly, a computerized diagnosis and operation registry was searched to identify all CABG procedures performed in the study period based on the Nordic Medico-Statistical Committee (NOMESCO) surgical classification system. Secondly, a centralized cardiac surgery database was examined to ensure matching of the procedures identified in the former registry. Following patient identification, clinical data were manually gathered from patient charts and surgical reports from Landspitali, as well as medical records from all other hospitals in Iceland treating patients for cardiovascular disease.

### Determining NISD

As non-infectious sternal dehiscence was our main focus, all patients with evidence of DSWI were excluded (n = 20, 0.87%) based on The Centers for Disease Control (CDC) criteria for post-sternotomy mediastinitis [12], giving us our final study population 2280 patients. For the remaining patients, NISD was determined by identifying patients diagnosed postoperatively or requiring re-admission after discharge due to clinical signs of sternal dehiscence. All NISD cases were re-evaluated for negative pre- and post-operative mediastinal or wound cultures taken before and during re-operation.

### Clinical variables and definitions

Documented demographics included patient baseline characteristics, comorbidities, risk factors for cardiovascular disease, and prior cardiac history (i.e. history of arrhythmia, heart failure, myocardial infarction, or percutaneous coronary intervention). COPD was defined as a history of emphysema or chronic bronchitis as documented in patients’ charts, and in most cases confirmed with lung function tests preoperatively. Impaired kidney function was defined as a preoperative estimated glomerular filtration rate (eGFR) < 60 ml/min/1.73m^2^, regardless of the stage of albuminuria. Clinical symptoms of heart failure and angina were classified according to the New York Heart Association (NYHA) functional classification [13] and Canadian Cardiovascular Society (CCS) grading system [14], respectively. As almost all patients had received a preoperative echocardiograph, we could estimate the 30-day mortality by calculating the EuroSCORE II [15]. In case of missing preoperative reports, the intraoperative echocardiography was used. Finally, preoperative diagnostic angiographies were used to estimate the extent and severity of coronary artery disease.

For intraoperative variables, we described the cross-clamp time, operative technique used, the number of distal anastomoses, the proportion of left internal mammary artery (LIMA) grafts, and whether the surgery was emergent (occurring within 24 h from hospital admission), semi-acute (occurring in the same hospital stay) or elective. Additionally, we described the prevalence of off-pump CABG, and whether an intra-aortic balloon pump (IABP) was used intraoperatively or in the immediate postoperative period. Finally, postoperative bleeding was documented with chest tube output within 24 h, and the total length of hospital stay in days, as well as the length of ICU stay in days, was described for all patients.

All patients were followed until December 31, 2020, and no patient was lost to follow-up. The median follow-up time was 9.5 years, IQR [5.6; 13.3].

### Statistical analysis

Statistical analyses were carried out using Microsoft Excel (2019) and R (R Foundation for Statistical Computing, Austria), version 4.1.0, via R Studio (RStudio, PBC, USA), version 1.4.1103. Categorical variables were presented with count (%) and comparisons between groups made using the *χ*^2^ test or Fisher’s exact test for expected values less than 5. Continuous variables were presented with mean (SD) or median (IQR) and comparisons between groups made using the t-test or Wilcoxon rank-sum test, as appropriate. The crude incidence of NISD was computed as a proportion of total CABG procedures performed annually. A Poisson regression model with procedure volume as an offset was used to estimate temporal changes in the incidence of NISD as a proportion of annual procedures. The time to diagnosis of NISD, as well as five-year all-cause mortality, were graphically presented with the Kaplan–Meier method.

## Results

**Fig. 1:**
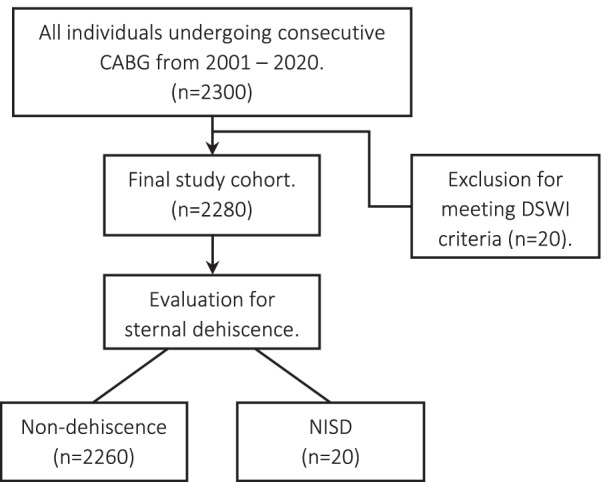
A flowchart showing patient inclusion for the present study

A total of 2300 patients underwent CABG over the course of the study. After the exclusion of 20 patients with documented DSWI, our final study cohort consisted of 2280 patients, with 20 (0.88%) of those having NISD and all of them being reoperated for that (Fig. 1).

**Fig. 2:**
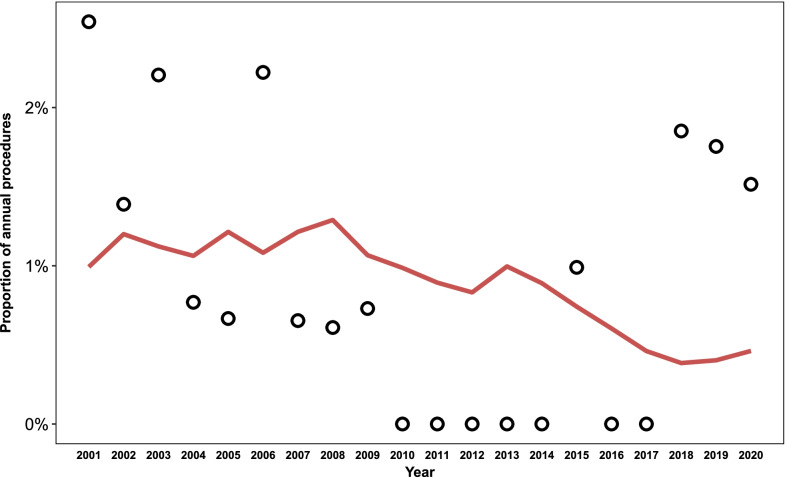
Incidence of non-infectious sternal dehiscence as a proportion of annual CABG procedures. The incidence did not change significantly over the study period

**Fig. 3:**
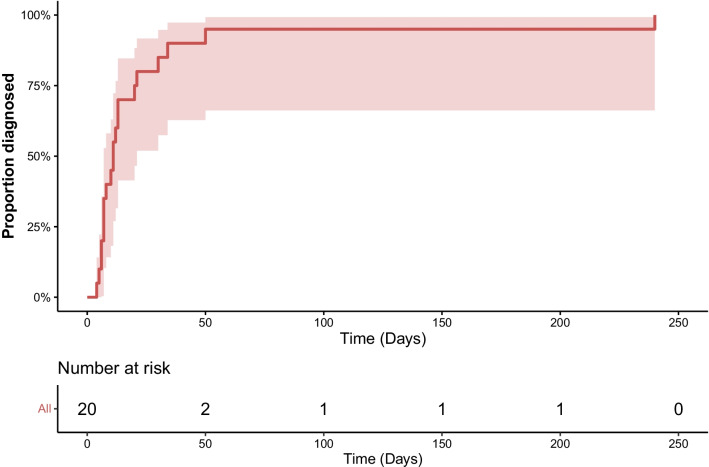
A cumulative Kaplan–Meier graph depicting the time to diagnosis of non-infectious sternal dehiscence

The average incidence of NISD over the study period was 0.88% of annual procedures, with 16 and 4 of the cases occurring in the first and last half of the study period, respectively. Figure 2 depicts the crude incidence of NISD as a proportion of annual CABG procedures. There was not a significant decline in the annual incidence of NISD over the study period (0.97%, 95% CI [− 1.4%, 0.59%], p for trend = 0.98). The median time to diagnosis and re-operation from the index surgery was 12 days, ranging from 4 to 240 days, with 17 (85%) of the re-operations occurring in the same hospital stay and three (15%) occurring after hospital discharge (Fig. 3).

Table 1 shows a comparison of patient characteristics and intra-operative variables between the two groups. Compared to patients without evidence of sternal dehiscence, NISD patients were significantly older (72 vs. 67 years, p = 0.02), had a higher BMI (31.4 vs. 27.8 kg/m^2^, p = 0.004), and lower LVEF (50% vs. 55%, p = 0.001). Patients with NISD were more likely to have a diagnosis of COPD (25.0% vs. 7.2%, p = 0.009), a history of MI (60.0% vs. 24.2%, p = 0.001), diabetes (35.0% vs. 18.3%, p = 0.10), and a prior hospitalization for heart failure (20.0% vs. 8.1%, p = 0.13). Accordingly, they were also more likely to have worse preoperative symptoms (NYHA and CSS class IV) and a higher EuroSCORE II (2,5 vs. 1,4, p = 0.009). Intraoperative variables were comparable for both groups, although NISD patients had a higher frequency of intra-aortic balloon pump (IABP) used intraoperatively or in the postoperative period (20.0% vs. 4.2%, p = 0.004).

**Table 1:** Patient demographics and operative parameters in both groups

Variables	Intact sternum (n = 2260)	NISD (n = 20)	p value
Age, years (Mean, SD)	67 (9.3)	72 (8.9)	0.02
Female	394 (17.4)	4 (20.0)	0.99
BMI, kg/m^2^	27.8 [25.4, 30.9]	31.4 [28.0, 34.6]	0.004
History of Smoking	1555 (68.9)	16 (80.0)	0.40
Hypertension	1483 (65.9)	15 (75.0)	0.54
Diabetes	411 (18.3)	7 (35.0)	0.10
Hyperlipidemia	1224 (55.6)	9 (45.0)	0.47
COPD	162 (7.2)	5 (25.0)	0.009
History of MI	546 (24.2)	12 (60.0)	0.001
History of cardiac arrhythmia	258 (11.4)	1 (5.0)	0.58
Heart failure	184 (8.1)	4 (20.0)	0.13
Valvular disease	82 (3.6)	1 (5.0)	1.0
Previous PCI	533 (23.6)	6 (30.0)	0.69
Hemoglobin, g/L	142 [132, 151]	143 [136, 147]	0.99
eGFR, ml/min/1.73m^2^	76.4 (20.4)	69.8 (19.8)	0.15
EuroSCORE II	1.4 [0.9, 2.5]	2.5 [1.3, 5.6]	0.009
CSS class 4	907 (40.1)	13 (65.0)	0.04
NYHA class IV	385 (17.0)	7 (35.0)	0.07
LVEF, %	55 [50, 60]	50 [40, 55]	0.001
3-vessel disease	2018 (89.3)	20 (100.0)	0.24
Left main stem disease	944 (41.8)	8 (40.0)	1.0
Off-pump CABG	401 (17.7)	3 (15.0)	0.98
Acute or semi-acute surgery	1238 (54.8)	11 (55.0)	0.99
ECC time, min	87 [71, 107]	90 [77, 98]	0.93
Cross-clamp time, min	46 [37, 58]	42 [37, 49]	0.09
LIMA-graft used	2134 (94.5)	18 (90.0)	0.70
Number of distal anastomoses	3.4 (0.9)	3.6 (0.8)	0.44
IABP used intra- or postoperatively	96 (4.2)	4 (20.0)	0.004

Number (%) or means with standard deviation

Values are presented as count (%) or median [IQR] unless specified otherwise

*BMI* body mass index; *eGFR*; estimated glomerular filtration rate; *CSS* Canadian Cardiovascular Society; *NYHA* New York Heart Association; *COPD* chronic obstructive pulmonary disease; *MI* myocardial infarction; *PCI* percutaneous coronary intervention; *ECC* extracorporeal circulation; *LIMA* left internal mammary artery; *IABP* intra-aortic balloon pump

A comparison of transfusions and length of stay for both groups is shown in Table 2. There was no difference in bleeding rates by chest tube output at 24 h postoperatively, however, patients in the NISD group more often received blood transfusions postoperatively. Additionally, the post-operative stay in the intensive care unit, as well as total hospital stay, was significantly longer in the NISD group (2 vs. 1 and 21 vs. 8 days respectively, p < 0.001 for both).

**Table 2:** Comparison of postoperative bleeding, blood products administrated, and the length of stay

Post op variables	Intact sternum (n = 2260)	NISD (n = 20)	p value
Chest tube output within 24 h., mL	730 [520, 1040]	623 [458, 1036]	0.43
Packed red cells administered, units	1 [0, 3]	2 [1, 8]	0.02
Length of ICU stay, days	1 [1]	2 [1, 5]	< 0.001
Total length of stay, days	8 [7, 11]	21 [17, 28]	< 0.001

Values are presented as median [IQR] unless specified otherwise.

*ICU *intensive care unit

Early postoperative complications (< 30 days) are shown in Table 3 for both groups. There were no observed differences for major complications, but NISD patients more often had infections in the leg after vein harvesting (30% vs. 10%, p = 0.006) and post-operative pneumonia (25% vs. 7%, p = 0.004).

**Table 3:** Comparison of minor and major early postoperative complications between groups

	Intact sternum (n = 2260)	NISD (n = 20)	p value
*Minor complications*			
New onset atrial fibrillation	857 (37.9)	11 (55.0)	0.18
Pleural effusion requiring drainage	287 (12.7)	4 (20.0)	0.52
Pneumonia	146 (6.5)	5 (25.0)	0.004
Urinary tract infection	71 (3.1)	2 (10.0)	0.27
Superficial infection of leg after vein harvesting	221 (9.8)	6 (30.0)	0.006
*Severe complications*			
Re-exploration for bleeding	114 (5.0)	2 (10.0)	0.62
Perioperative stroke	25 (1.1)	0 (0)	1.0
Multiorgan failure	62 (2.7)	2 (10.0)	0.19
Perioperative MI	91 (4.0)	1 (5.0)	1.0
30 day mortality	52 (2.3)	1 (5.0)	0.96

Values are presented as count (%) unless specified otherwise.

*MI* myocardial infarction

**Fig. 4:**
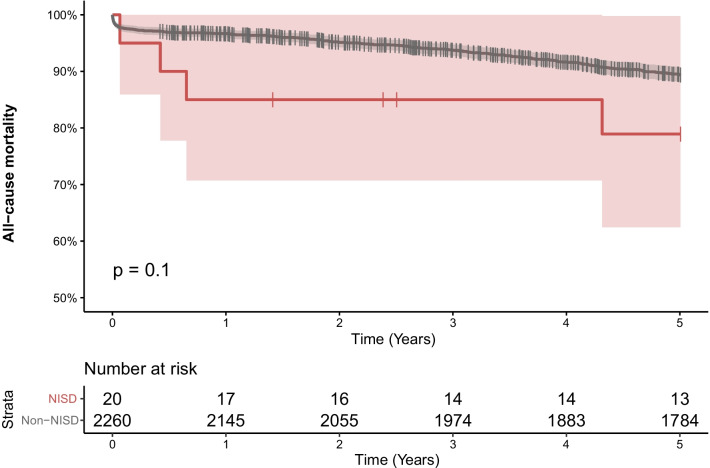
A Kaplan–Meier graph depicting the estimated overall survival with 95% confidence intervals (shades) of patients with non-infectious sternal dehiscence versus non-dehiscence

There were no significant differences in 30-day or 90-day mortality between the study groups, being 2.3% vs. 5.0% and 2.7% vs. 5.0% (p = 0.96 and 0.43 respectively). Similarly, there was no significant difference in mortality at 5 years from surgery (20.0% vs. 10.1%, p = 0.1) (Fig. 4).

## Discussion

In this retrospective whole-nation study with over 2000 CABG patients we found that the incidence of NISD was low (within 1%), and in line with previous studies. Although NISD patients were older and had more disease burden preoperatively, the differences in 30-day and 5-year mortality were not statistically different between the groups. Still, NISD should be considered a serious complication, reflected in over two weeks extension of hospital stay on average. Therefore, early diagnosis or prevention of NISD is important, both to reduce hospital costs and morbidity, and should especially be regarded in elderly patients, those with COPD, and patients with increased BMI.

An incidence of 0.88% is in line with previous studies describing NISD [1, 6]. However, studies reporting the incidence of NISD after open heart surgery are scarce, with some studies only including CABG patients [16], while others observe valve or a combination of CABG and valve procedures [1]. Although the incidence of NISD trended downwards in the latter half of the twenty-year study period, the difference was not statistically significant. It should be noted that the present study is underpowered to prove small to moderate changes in incidence. This is primarily due to the rarity of NISD but similarly influenced by the observed decline in CABG procedures over the last decades of the study.

The diagnosis of NISD was often delayed, occurring at a median of 12 days postoperatively. These patients usually have a slow recovery after surgery, with more pain issues than normally and serous fluid discharge from the sternotomy wound. As the definite clinical diagnosis of NISD with palpation is challenging, especially in obese patients, a more precise computed tomography (CT) scan may be indicated. Following the diagnosis of NISD, our patients were always taken directly to the operating room where the wound was inspected for potential infection and cultures (both swab and tissue samples) taken to rule out DSWI-induced dehiscence. None of the NISD patients had positive cultures and for the diagnosis of DSWI, the CDC criteria were followed [12].

Many of the factors associated with NISD resemble those for DSWI [7, 8], as presented in our primary analysis (Table 1). Factors such as advanced age, BMI, and notably COPD had the strongest association with NISD, and have previously been reported in this regard [6, 9, 10]. A possible explanation for these concurring results could be that a higher BMI and COPD, commonly associated with chronic coughing, may induce mechanical stress on the sternotomy closure. Similarly, older patients may be more prone to poor wound closure. Individually or in coexistence, these factors propose a conceivable mechanism in which the risk of NISD is increased by impairing the structural integrity of the wound closure. As previously stated, the study is however considerably underpowered to prove this correlation.

As an early diagnosis of NISD is imperative, it is beneficial to know potential risk factors of NISD in order to avoid the development of a much more serious DSWI. Prevention is also important, but most studies are underpowered, and large, randomized studies lacking [17]. In most institutions like ours, single stainless-steel wires are used for sternum closure where the sutures are passed around the sternal body and through the manubrium or sternum body. The optimal number of single wires is not known [18, 19], but Friberg et al. demonstrated that the use of fewer than six wires rather than seven or more increased the rate of sternal wound infection from 0.4 to 4.2% [20]. Cadaveric studies have indicated increased stability of the sternum by using double wires, especially in obese patients, and a more recent review and meta-analysis illustrated the same [21]. This also applies to the use of a figure-of-eight pattern for wire closure compared to single wires. Although Almdahl et al [1]. also showed a significantly lower ratio of NISD in 7835 patients closed with figure-of-eight compared to the single wire technique (0.066% vs. 0.66%), a meta-analysis including 111 studies did not prove any superiority of such a technique [22]. Furthermore, large randomized studies are lacking on the routine use of the Robicsek technique, or more recently the use of metal plates, sternal bands, or polymer cable ties for sternal fixation [17].

### Limitations and strengths

A strength of this study is that it includes all patients who underwent CABG in a well-defined population-based cohort, where all patients were operated and re-operated on in the same institution, a tertiary-care university hospital. This makes our findings less affected by a potential selection bias and treatment variability. Furthermore, by using centralized registries we could assess mortality data that was 100% complete, which makes our long-term survival data accurate. Similarly, our dataset of clinical variables was detailed and the median follow-up of 9.5 years median must be regarded as long.

The major limitation is the retrospective design and, therefore, inevitable missing data on factors that rely on the accuracy of registration. The number of NISD cases was also low, which limits our possibility to detect differences between NISD and patients with an intact sternum. Lastly, data on the number of steel wires used, single or double wire and figure-of-eight were not detailed enough to be presented.

## Conclusions

We found that the incidence of NISD was low (< 1%) after CABG, with both short- and long-term survival of NISD patients being comparable to patients with an intact sternum. Still, NISD is a serious complication, as reflected in the extended hospital stays of these patients. Therefore, early diagnosis or prevention of NISD is important, and should especially be regarded in elderly patients, those with COPD, and patients with increased BMI.

## Data Availability

The datasets generated and analyzed during the present study are not publicly available due to the privacy of the individuals and would violate the terms of the National Bioethics Committee of Iceland. The data may be shared on reasonable request to the corresponding author with the permission of the National Bioethics Committee of Iceland.

## Abbreviations

BMI: Body mass index
CABG: Coronary artery bypass grafting
CCS: Canadian Cardiovascular Society
CDC: Center for Disease Control
COPD: Chronic obstructive pulmonary disease
CPB: Cardiopulmonary bypass
DSWI: Deep sternal wound infection
eGFR: Estimated glomerular filtration rate
IABP: Intra-aortic balloon pump
ICU: Intensive care unit
IQR: Interquartile range
LIMA: Left internal mammary artery
LVEF: Left ventricular ejection fraction
MI: Myocardial infarction
NISD: Non-infectious sternal dehiscence
NOMESCO: Nordic Medico-Statistical Committee
NYHA: New York Heart Association
SD: Standard deviation
